# Ischemic stroke demographics, clinical features and scales and their correlations: an exploratory study from Jordan

**DOI:** 10.2144/fsoa-2022-0017

**Published:** 2022-08-05

**Authors:** Khaled Z Alawneh, Majdi Al Qawasmeh, Liqaa A Raffee, Abdel-Hameed Al-Mistarehi

**Affiliations:** 1 Department of Diagnostic Radiology & Nuclear Medicine, Faculty of Medicine, Jordan University of Science & Technology, Irbid, 22110, Jordan; 2 Department of Neurology, Faculty of Medicine, Jordan University of Science & Technology, Irbid, 22110, Jordan; 3 Department of Accident & Emergency Medicine, Faculty of Medicine, Jordan University of Science & Technology, Irbid, 22110, Jordan; 4 Department of Public Health & Family Medicine, Faculty of Medicine, Jordan University of Science & Technology, Irbid, 22110, Jordan

**Keywords:** Barthel Index, BI, gender, hyperlipidemia, hypertension, ischemic stroke, modified Rankin Scale, mRS, NIHSS, risk factors, stroke

## Abstract

**Aims:**

The authors aimed to assess the ischemic stroke risk factors and scales.

**Materials & methods:**

A retrospective cohort study was conducted on patients with acute ischemic stroke (from January 2017 to December 2018). The scores of the National Institutes of Health Stroke Scale (NIHSS) at admission and discharge and of the modified Rankin Scale (mRS) and Barthel Index (BI) scale post-month of the stroke were collected.

**Results:**

Out of 376 patients, 359 were included, with a mean (standard deviation) age of 67.8 (12.2) years and male predominance (56.2%). Hyperlipidemia and hypertension were the most prevalent comorbidities (91.1% and 80.5%, respectively). The NIHSS, BI and mRS scores were worse among women, with no significant effects for comorbidities. The NIHSS scores at admission and discharge were significantly correlated with the post-month BI and mRS scores.

**Conclusion:**

The study findings suggest a complex interplay of gender, strict control and prevention of the modifiable stroke risk factors, as well as the association of neurological deficits' intensity with the functional outcomes.

## Plain language summary

This study aimed to explore the demographics, the clinical risk factors and the scores of the National Institutes of Health Stroke Scale (NIHSS), modified Rankin Scale (mRS) and Barthel Index (BI) scale at different points of time among the survivors of acute ischemic stroke at a tertiary hospital in Jordan. Also, the study aimed to investigate the differences in the scales' scores by the patients' characteristics and the correlations between these scales. Out of 376 screened patients, 359 were included. Their mean (standard deviation) age was 67.8 (12.2) years, and 56.2% were men. Compared with male participants, women scored significantly worse on the NIHSS at admission (7.61 [5.51] vs 9.47 [6.64]; p = 0.048), NIHSS at discharge (5.57 [4.72] vs 7.40 [5.88]; p = 0.028) and BI scale 1 month post-event (78.68 [28.33] vs 66.03 [35.86]; p = 0.011). The mean (standard deviation) mRS score post-month of stroke was lower in men (2.4 [1.7]) than in women (2.9 [1.9]), with a lack of statistical significance (p = 0.097). Thus, despite the male predominance in the cohort, women tended to have a more severe stroke, worse neurological impairment and poorer functional outcomes. Hyperlipidemia had the highest prevalence, sensitivity, positive predictive value and negative predictive value rates, followed by hypertension. No statistically significant differences existed in the comorbidities' NIHSS, BI scale and mRS scores. Strong and significant correlations were observed between the scores of NIHSS at admission and discharge and the BI scale and mRS scores at 1 month post-event. Thus, the authors concluded that neurological deficit severity has a potential role in predicting functioning outcomes and *vice versa*.

## Graphical abstract

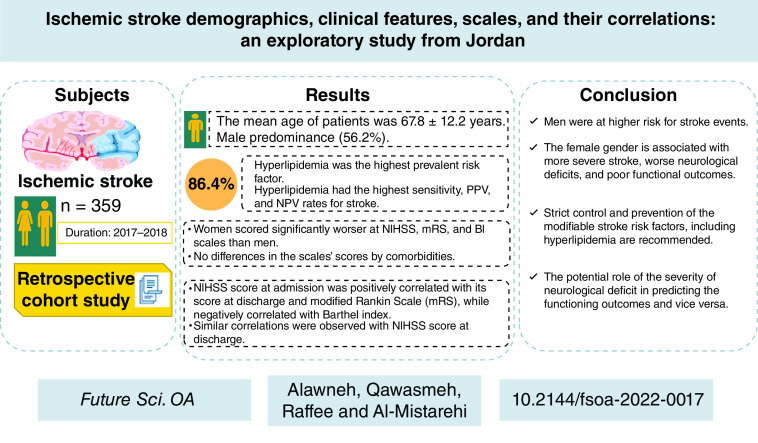

Stroke is a vascular insult characterized by a loss of blood flow to part of the brain, which leads to an acute brain injury and neurological impairment. Per the WHO definition, stroke is progressively developed clinical findings of focal disturbance of cerebral function with symptoms lasting 24 h or longer, or leading to death, with no apparent cause other than of vascular origin [1]. Globally, stroke is one of the leading causes of disability and is the second leading cause of death worldwide [2]. Several modifiable and nonmodifiable risk factors could lead to acute stroke events [3,4]. Higher stroke risk is associated with older age and the male gender, and other risk factors are established from health conditions, including hyperlipidemia, diabetes mellitus (DM), hypertension (HTN) and cardiovascular diseases [5,6]. Also, stroke can be attributed to smoking, alcohol consumption, obesity and hyperhomocysteinemia [7–10]. Globally, the prevalence rate of stroke for women is lower than for men [6]. Ischemic stroke has been associated with a lack of exercise, DM, HTN and hyperlipidemia [11]. In contrast, hemorrhagic stroke has been substantially related only to HTN [11]. Dementia and depression are other risk factors associated with stroke [12].

The prevalence estimates of stroke in low- and middle-income countries have doubled over the last 40 years, while stroke prevalence has decreased by 42% in high-income countries [13–15]. In Middle Eastern countries, the prevalence rates of stroke are variable, rapidly becoming a major dilemma in the region [16,17]. Similarly, a variable burden of stroke has been experienced in the Middle East region. The prevalence estimates for all strokes range between 22.7 and 250 per 100,000 individuals in this region per year [16]. Over the past few decades, the lifestyle has changed rapidly, which has caused a transition to a high prevalence of stroke due to the dramatic modification of the economic, environmental and social conditions in this region. Previous studies in Saudi Arabia provided hospital-based crude annual prevalence rates of stroke, accounting for 43.8 per 100,000 individuals in Riyadh, 29.8 per 100,000 individuals in the eastern province of Saudi Arabia and 15.1 per 100,000 individuals in Jizan [18,19]. An overall minimal prevalence rate of hospitalized first-time stroke of 57.64 per 100,000 individuals per year is reported in the Aseer region of Saudi Arabia [18,19].

Implementing enhanced risk prediction of stroke is still challenging due to the modest effect size of genetic risk variations, and it remains a distant goal. However, with the significant rise of the number of variations discovered in stroke risk factors at big, multinational consortia, these factors could significantly improve the risk classification of patients. Several tools are available for estimating stroke severity and outcomes in the hospital. One of these standard scales is the National Institutes of Health Stroke Scale (NIHSS), a strong outcome predictor for stroke with a high predictive value [20]. However, when later variations affect the patient's trajectory, the impact of early NIHSS measures on the final result is diminished [21]. The modified Rankin Scale (mRS) and the Barthel Index (BI) scale are the other two commonly used functional impairment and disability measures that have been shown to be accurate and reliable in predicting stroke outcomes [21]. However, there is interobserver variability in mRS assessment between countries [22]. Inter-rater variability brings noise into trial outcome evaluations, lowering the clinical trial power to identify treatment outcomes [23]. Most of the available literature in this regard is from Western countries, yet local studies from the developing countries are scant. The application of NIHSS, mRS and BI scale scores in patients with stroke could have clinical utility and be helpful for the prognostic objectives. Thus, this study aimed to investigate the demographics, clinical characteristics and stroke risk factors among the survivors of patients admitted with acute ischemic stroke at a tertiary university hospital in the north of Jordan. Also, it aimed to measure the scores of the NIHSS, mRS and BI scale at different points in time. In addition, this study aimed to assess the differences among the stroke scales by ischemic stroke patients' characteristics and to investigate the correlations among these scales regardless of the stroke treatment modality.

## Materials & methods

### Study design, settings, procedure & sample

A retrospective cohort study was carried out at King Abdullah University Hospital, a tertiary hospital in the north of Jordan affiliated with the Jordan University of Science and Technology. The authors reviewed the medical records of all patients admitted to the hospital with acute ischemic stroke from January 2017 to December 2018. The hospital patient records were registered using the International Statistical Classification of Diseases and Related Health Problems, 10th revision, diagnosis standards.

The history and physical examination of patients were reviewed. Demographics, presenting speech symptoms and comorbidities, including HTN, DM, hyperlipidemia and atrial fibrillation, were extracted from patients' electronic medical records. The NIHSS scores for each patient at admission and discharge were collected. The NIHSS assesses the patient's consciousness level, visual-field loss, extraocular movements, motor strength, ataxia, sensory loss, language, dysarthria and neglect [24–26]. In the NIHSS, the higher the score, the more the stroke patient is neurologically impaired and disabled and has poor outcomes [27,28]. Also, BI scale and mRS scores 1 month after the stroke event were calculated for each patient. The BI scale and mRS are commonly used for measuring the extent and degree of functioning independently and mobility in activities of daily living (ADLs) [29–33]. The BI total score ranges from 0 to 100, and a higher BI score reflects a more remarkable ability to function independently. On the other hand, the mRS total score ranges between 0 and 6, where the score of 0 represents no disability, the 5 score is disability requiring constant care and the score of 6 represents death. Trained neurologists rated the patient's ability to answer the questions and perform the activities and, thus, calculated the scores on scales.

**Figure 1.**
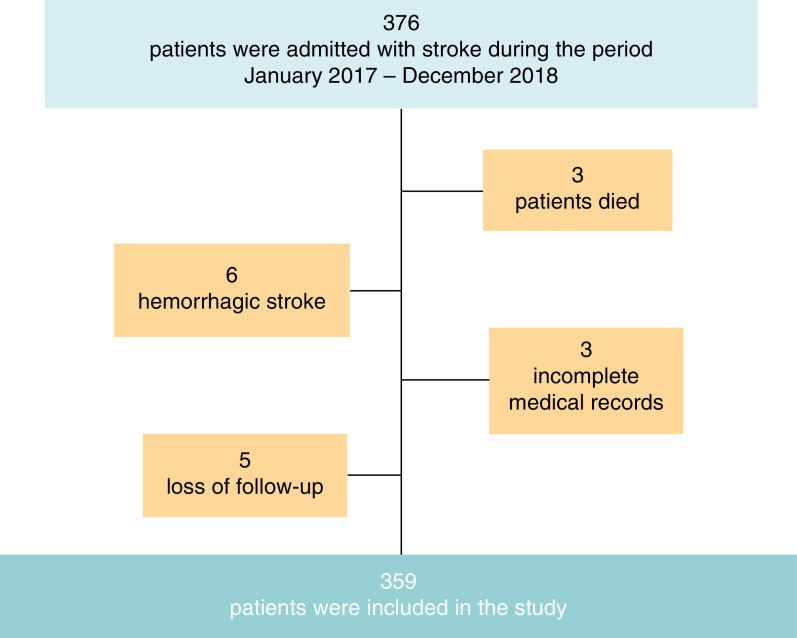
Flowchart of the subjects' enrollment in the study.

The adult patients admitted with acute ischemic stroke during the study period and who survived after the stroke event were eligible for participation in this study. Exclusion criteria included those younger than 18 years old, patients with hemorrhagic stroke, those with significant missing data in their medical records and loss of follow-up. Thus, out of 376 screened patients, 17 were excluded because of death, hemorrhagic stroke diagnosis, missing data regarding the scales' components in their medical records or loss of follow-up. Thus, the number of consecutive surviving patients who fulfilled the inclusion and exclusion criteria during the allocated period and were included in this study was 359 patients with acute ischemic stroke (Figure 1).

### Statistical analysis

IBM SPSS software, Windows version 24, was used to analyze the data. The continuous variables were presented in mean and standard deviation (SD), while the categorical variables were displayed in frequencies and percentages. A Chi-square test was applied to assess the categorical variables, while an independent *t*-test was used to observe the mean difference in the continuous variables between two groups of patients. P ≤ 0.05 was considered statistically significant. The sensitivity, specificity, positive predictive value (PPV) and negative predictive value (NPV) were calculated for comorbidities among the patients with ischemic stroke. The 95% CIs were reported.

## Results

Of 376 patients diagnosed with acute ischemic stroke, 359 survivors were included in the final analysis. The mean (SD) age of participants was 67.8 (12.2) years, and more than half of the patients were men (56.2%). Hyperlipidemia and HTN were the most common comorbidities among the patients, with prevalence rates of 91.1% and 80.5%, respectively. Approximately half of the patients (50.2%) had DM. Dysarthria was the most common speech abnormality (75.8%). The demographic and clinical characteristics of study participants are depicted in Table 1.

**Table 1.** Demographic and clinical characteristics of study participants (n = 359).

Variable		Frequency	Percentage
Age (years), mean ± SD = 67.8 ± 12.2			
Gender	Male	202	56.2
	Female	157	43.8
Comorbidities	Hypertension	289	80.5
	Diabetes mellitus	187	52.1
	Hypertension + diabetes mellitus	161	44.9
	Hyperlipidemia	327	91.1
	Atrial fibrillation	30	8.4
Speech abnormality	Dysarthria	272	75.8
	Aphasia	76	21.2
	Aphasia + dysarthria	9	2.5
	Anarthria	2	0.6
National Institutes of Health Stroke Scale score at admission, mean ± SD = 8.4 ± 6.0			
National Institutes of Health Stroke Scale score at discharge, mean ± SD = 6.3 ± 5.3			
Barthel Index scale score 1 month post-stroke event, mean ± SD = 73.1 ± 32.3			
Modified Rankin Scale score 1 month post-stroke event, mean ± SD = 2.6 ± 1.8			
Modified Rankin Scale score 1 month post-stroke event:	0	53	14.8
	1	81	22.6
	2	40	11.1
	3	42	11.7
	4	75	20.9
	5	63	17.6
	6	5	1.4

SD: Standard deviation.

Overall, the mean (SD) NIHSS scores at admission and discharge were 8.4 (6.0) and 6.3 (5.3), respectively. The mean (SD) scores of BI and mRS 1 month after the stroke event were 73.1 (32.3) and 2.6 (1.8), respectively. The differences among the NIHSS, BI and mRS scores by patients' gender and comorbidities are shown in Table 2. Men had significantly lower mean (SD) NIHSS scores at admission (7.61 [5.51]) and discharge (5.57 [4.72]) than women (9.47 [6.64] and 7.40 [5.88], respectively) with p-values of 0.048 and 0.028, respectively. Similarly, the mean (SD) mRS score 1 month post-stroke event was lower in male participants (2.4 [1.7]) than in female patients (2.9 [1.9]), but this difference was not statistically significant (p = 0.097). In contrast, the mean (SD) BI score 1 month after stroke event was significantly higher among male participants (78.68 [28.33]) than female patients (66.03 [35.86]) (p = 0.011). However, there were no statistically significant differences in the NIHSS, BI and mRS scores by patients' comorbidities, including HTN, DM, HTN + DM, hyperlipidemia and atrial fibrillation (p > 0.05).

**Table 2.** Differences in the scores of the National Institutes of Health Stroke Scale, Barthel Index scale and modified Rankin Scale by gender and comorbidities.

			Mean	SD	p-value
Gender	NIHSS score at admission	Male	7.61	5.51	0.048
		Female	9.47	6.64	
	NIHSS score at discharge	Male	5.57	4.72	0.028
		Female	7.40	5.88	
	BI score 1 month post-stroke	Male	78.68	28.33	0.011
		Female	66.03	35.86	
	mRS score 1 month post-stroke	Male	2.39	1.73	0.097
		Female	2.85	1.85	
Hypertension	NIHSS score at admission	No	8.85	6.88	0.658
		Yes	8.32	5.89	
	NIHSS score at discharge	No	6.03	4.83	0.695
		Yes	6.45	5.44	
	BI score 1 month post-stroke	No	72.12	34.05	0.834
		Yes	73.44	32.04	
	mRS score 1 month post-stroke	No	2.61	2.03	0.963
		Yes	2.59	1.74	
Diabetes mellitus	NIHSS score at admission	No	8.61	6.14	0.382
		Yes	7.82	5.36	
	NIHSS score at discharge	No	6.75	5.62	0.335
		Yes	5.95	4.91	
	BI score 1 month post-stroke	No	74.73	31.59	0.880
		Yes	73.86	30.99	
	mRS score 1 month post-stroke	No	2.53	1.79	0.880
		Yes	2.57	1.75	
Hypertension + diabetes mellitus	NIHSS score at admission	No	8.76	6.27	0.281
		Yes	7.78	5.36	
	NIHSS score at discharge	No	6.75	5.57	0.296
		Yes	5.89	4.89	
	BI score 1 month post-stroke	No	73.67	32.13	0.956
		Yes	73.95	31.27	
	mRS score 1 month post-stroke	No	2.54	1.80	0.993
		Yes	2.55	1.73	
Hyperlipidemia	NIHSS score at admission	No	7.50	5.78	0.722
		Yes	8.25	5.81	
	NIHSS score at discharge	No	5.38	5.48	0.588
		Yes	6.42	5.32	
	BI score 1 month post-stroke	No	70.00	38.36	0.710
		Yes	74.27	31.26	
	mRS score 1 month post-stroke	No	2.70	2.26	0.845
		Yes	2.59	1.77	
Atrial fibrillation	NIHSS score at admission	No	8.03	5.44	0.276
		Yes	9.85	8.69	
	NIHSS score at discharge	No	6.13	4.91	0.127
		Yes	8.46	8.40	
	BI score 1 month post-stroke	No	74.59	30.73	0.586
		Yes	69.62	39.82	
	mRS score 1 month post-stroke	No	2.33	1.91	0.547
		Yes	2.58	1.75	

BI: Barthel Index; mRS: modified Rankin Scale; NIHSS: National Institutes of Health Stroke Scale; SD: Standard deviation.

Table 3 summarizes the linear correlations between the different scales using Pearson correlation, with its coefficient (r). The NIHSS score at admission was positively correlated with the NIHSS score at discharge (r = 0.952; p < 0.001) and with the mRS score 1 month after the stroke event (r = 0.765; p < 0.001). Also, a strong but negative correlation was found between NIHSS score at admission and BI score 1 month post-stroke event (r = -0.747; p < 0.001). Similarly, the NIHSS score at discharge was positively correlated with the mRS score and negatively with the BI score. Also, a negative association was observed between mRS and BI scores 1 month post-stroke event (r = -0.832; p < 0.001).

**Table 3.** Pearson correlations (r) of scales among the study participants.

		NIHSS score at discharge	Barthel Index score 1 month post-stroke event	Modified Rankin Scale score 1 month post-stroke event
NIHSS score at admission	r	0.952	‐0.747	0.765
	p-value	<0.001	<0.001	<0.001
NIHSS score at discharge	r	1	‐0.740	0.744
	p-value	–	<0.001	<0.001
Barthel Index score 1 month post-stroke event	r	‐0.740	1	‐0.832
	p-value	<0.001	–	<0.001

NIHSS: National Institutes of Health Stroke Scale.

Among the studied comorbidities, hyperlipidemia had the highest sensitivity for stroke, with a value of 86.4% (95% CI: 75.3–93.4), followed by 79.2% (95% CI: 63.5–91.1) for HTN. The highest specificity was measured at 94.3% (89.9–98.9%) for HTN and 90.3% (88.9–96.8%) for hyperlipidemia. The highest PPV and NPV values were measured for hyperlipidemia, with 92.6% (95% CI: 85.2–97.5) and 93.5% (95% CI: 88.9–99.4), respectively (Table 4).

**Table 4.** Validation analysis of comorbidities among patients with ischemic stroke.

Validation parameters	Comorbidities, percentage (95% CI)				
	Hyperlipidemia	Hypertension	Diabetes mellitus	Hypertension + diabetes mellitus	Atrial fibrillation
Sensitivity	86.4% (75.3–93.4)	79.2% (63.5–91.1)	74.6% (58.7–90.5)	75.8% (60.1–89.4)	52.8% (40.5–63.4)
Specificity	90.3% (88.9–96.8)	94.3% (89.9–98.9)	89.7% (72.3–94.1)	88.9% (71.5–94.0)	76.5% (59.4–73.4)
Positive predictive value	92.6% (85.2–97.5)	87.9% (87.9–96.9)	79.2% (61.4–9.5)	61.2% (49.8–71.9)	56.9% (48.6–66.5)
Negative predictive value	93.5% (88.9–99.4)	83.1% (70.9–92.5)	76.4% (60.1–74.4)	76.7% (61.4–75.4)	60.1% (51.3–74.8)

## Discussion

This study evaluated the demographic and clinical characteristics of 359 survivors diagnosed with acute ischemic stroke at a tertiary hospital in the north of Jordan and investigated their associations with NIHSS, BI and mRS scores. The cohort observed male predominance (56%) and high prevalence rates of hyperlipidemia, HTN and DM comorbidities. Among these comorbidities, hyperlipidemia had the highest sensitivity, PPV and NPV values for stroke. Dysarthria was the most common speech abnormality. Despite the higher prevalence of ischemic stroke among men than women, the NIHSS, BI and mRS scores were better among men, with no statistically significant effects for comorbidities on the scores. Significant linear correlations were captured between the NIHSS scores at admission and discharge, and the BI and mRS scores at 1 month after the stroke event.

The prevalence rates of stroke have increased by 12% in low- and middle-income countries, resulting in considerable disability and mortality rates, with an anticipated increase in related deaths and the overall global burden of stroke [17,35]. The authors of the present study observed a male predominance in the cohort by 56% of patients, which approximates the Soliman *et al.* finding of 54% male prevalence among 167 patients with acute ischemic stroke recruited from a hospital in Egypt [36]. This finding also matches other studies from Germany and Iran in which male patients represented 58% and 55%, respectively [37,38]. Also, a study from the Arabian Gulf countries reported a male predominance among patients with ischemic stroke [39]. Men have a greater risk of developing ischemic stroke than women, which could be explained by hormonal constitutional variables, higher smoking rates and stressful conditions [40]. Previous studies indicated that stroke prevalence increased with advancing age, which is concordant with our observation in the present study that most patients with ischemic stroke were elderly, with a mean age of 67.8 years [40,41]. Over time, the cumulative impact of aging on the cardiovascular system and the increasing nature of stroke risk factors raise the risk of ischemic stroke [41].

In the current study, hyperlipidemia was documented in 91.1% of the patients with acute ischemic stroke as the most prevalent comorbidity, which is higher than the findings of previous studies, where hyperlipidemia was reported in 35–54% of stroke patients [36,37]. Also, previous clinical trials and registries reported that up to 60% of stroke patients have high blood lipids, such as serum cholesterol [42–44]. Elevated serum cholesterol levels above 7.0 mmol/l are associated with a higher risk of stroke [45]. The pathophysiology includes the disruption of endothelial and smooth muscle function, interruption of cerebral blood flow regulation, reduction of the collateral cerebral perfusion and coronary or cervical atherosclerosis, which prompts atherothrombotic and cardioembolic stroke [46]. However, compared with coronary or peripheral vascular disease, the potential significance of lipids as a stroke risk factor is less apparent due to the heterogeneity of stroke [47].

Previous studies reported controversial results on the influence of hyperlipidemia on stroke outcomes. Some investigations claimed protective effects of hyperlipidemia in stroke patients, primarily by reducing the mortality rates [48,49]. Others reported that hyperlipidemia is associated with a reduction in the white matter hyperintensity volume and leukoaraiosis in stroke patients, which could predict the stroke infarct progression leading to poor outcomes [48,50]. Moreover, Cohen *et al.* indicated a causality link between the abnormal serum levels of low-density lipoproteins and high-density lipoproteins and the observed reductions in white matter integrity among obese adults [51]. Moreover, some other studies showed the adverse effects of hyperlipidemia on the functional, neurological and survival outcomes in patients with stroke [52,53]. The significant impacts of lipid-lowering medications, such as HMG-CoA reductase inhibitors, in ameliorating neurological outcomes and lowering stroke incidence in various patient categories, including those with coronary heart disease, DM and HTN, as well as the elderly, were previously reported [54–57]. This knowledge supports these findings of the highest sensitivity of hyperlipidemia for stroke.

Previous studies reported HTN as a common risk factor for ischemic stroke, with prevalence estimates of 57–65% [58,59]. These rates are still lower than the estimated prevalence rate of HTN in the present study (80.5%). However, these findings are consistent with a previous study from the USA, which reported that 211,713 out of 276,734 patients with ischemic stroke were hypertensive (76.5%) [60]. Also, previous studies from Egypt, a Mediterranean country, reported HTN prevalence estimates of 83.1–88.5% among patients with ischemic stroke, which is concordant with the present study's finding [61,62]. In the present study, DM was recorded in 52% of patients, which is higher than in previous studies [37,38]. Atrial fibrillation was reported in 8% of this study's patients, which is lower than the previously reported rates by Saposnik *et al.* and Kim, which were 26% and 17%, respectively [63,64].

The role of gender as a significant risk factor for ischemic stroke incidence, progression and functional outcome was observed in this study. Although the men were at higher risk for stroke, the NIHSS scores at admission and discharge and the BI and mRS 1 month after the stroke event scores were better among men than women. Thus, the female gender was associated with more severe stroke, neurological deficits, poor outcomes and disabilities, agreeing with the findings of several studies [65–68]. In a previous clinical trial on acute ischemic patients treated with recombinant tissue plasminogen activator, men tended to have good functional outcomes three-times more than women [69]. This gender difference could be attributed to the postponed patient seeking of treatment, delayed hospital admission, not in-time access to the appropriate management, surviving spouse disadvantages, and less undergoing diagnostic stroke work-up among women with acute ischemic stroke than men [65,70,71]. Musa *et al.* reported a significant change in the mean (SD) of BI scores from 35.1 (39.4) at the time of discharge to 64.4 (39.5) at 1 month post-discharge, which is similar to the mean (SD) BI score in the present study's cohort (73.1 [32.3]) 1 month post-stroke event [72]. Moreover, the authors reported that the mean BI score was lower among women than men at the time of discharge (31.6 vs 41.8), which is concordant with the BI mean values in the present study's cohort [72].

This study did not find any statistically significant differences in the NIHSS, BI and mRS scores by the comorbidities. This finding is agreeable with a Phan *et al.* report on the minimal contribution of comorbidities, represented by the Charlson comorbidity index, in predicting the functional disability after ischemic stroke [73]. In contrast, other studies reported the comorbidities, such as HTN and atrial fibrillation, as significant predictors of stroke outcomes [63,64,74,75]. Kim *et al.* found that among stroke risk factors, atrial fibrillation was significantly correlated with NIHSS and mRS scores [64]. The lack of statistical significance for comorbidities in the present study's results could be attributed to the small sample size of patients with atrial fibrillation comorbidity. In contrast, the extremely high prevalence rates of hypertension and hyperlipidemia in the cohort could also limit the statistical significance.

Per previous studies, the findings of the current study proved that the NIHSS is a universal predictor of functional outcomes among patients with ischemic stroke [73,76,77]. NIHSS is the most commonly used tool for stroke-associated neurological deficits in routine clinical practice and clinical research [78]. Significant inverse correlations were depicted between the NIHSS and BI scores, which reflects the correlation between ADLs and cortical motor and cognitive functions [79–81]. Kwakkel *et al.* reported significant Spearman correlation coefficients between the BI score at 6 months and the NIHSS scores on day 2 (r_s_ = 0.549), day 5 (r_s_ = 0.592) and day 9 (r_s_ = 0.567) with a p-value of <0.001 for each correlation. The authors also captured a substantial positive link between NIHSS and mRS scores, reflecting the importance of neurological deficit severity in predicting functional disability outcomes after stroke. This finding is supported by the Saver *et al.* report of significant improvement in the correlation coefficients between the NIHSS scores over the observational period and the final 90-day mRS score, from 0.51 at 1–3 h to 0.72 at 1 day and to 0.87 at 90 days post-acute ischemic stroke event onset [82].

### Limitations of the study

This study has several limitations. First, the study is limited by the small sample size of the patients and being a single-center study. The second limitation is the short period of post-stroke follow-up, as the BI and mRS measurements were obtained once 1 month after the stroke event without more measurements. Thus, more measurements for extended follow-up periods are recommended to investigate the scores' changes on stroke scales, as the improvements could be extended until 12 months post-stroke [83]. Third, the study's retrospective nature tends to provide incomplete and missing data, as well as the considerable difficulty in achieving rapport and the paucity of visual clues in this design. Thus, multiple potential factors such as smoking [10,84], mental illnesses [85–87], stroke size and location [88] and laboratory findings [9,89–91] were not abstracted in this study due to missing such data in the medical records. A multicenter prospective study on a large sample of patients with ischemic stroke investigating all potential stroke risk factors over a follow-up period of at least 12 months is suggested. Fourth, the stroke scales were scored by different investigators; thus, potential bias in the results could not be ruled out, even though those investigators were trained neurologists and certified for neurological assessments of patients in line with the up-to-date guidelines. Finally, the study did not include other essential scales, such as the Katz Index, Frenchay Activities Index, Nottingham Extended ADL, Functional Ambulation Classification and Walking Handicap Scale, which could provide a better representation of the functional outcomes [92–95].

## Conclusion

The study findings reported the highest sensitivity, PPV and NPV values of hyperlipidemia for stroke events and proposed the high prevalence rate of hyperlipidemia, among other stroke risk factors, with over 90% of the cohort having hyperlipidemia. Thus, lifestyle, behavioral and pharmacological interventions are recommended to strictly control the modifiable stroke risk factors, such as hyperlipidemia, in the population. Also, preclinical investigations are invited to evaluate the influence of stroke risk factors, including hyperlipidemia, and different therapies on functional recovery in stroke patients while accommodating new neurorestorative strategies. This study sheds light on the importance of the primary prevention of dyslipidemia and the administration of lipid-lowering drugs in reducing stroke risk and its poor outcomes. The study findings suggest the complex interplay of gender in the cohort, with a stroke being more common among men, while female stroke patients were identified as a higher-risk subgroup for poor neurological deficits and functional disability outcomes. Studies with more detailed information are required to explore the gender role further. The comorbidities have no observed effects on stroke outcomes. Significant correlations were captured between the neurological impairment (NIHSS) and the ADLs performance ability and functional outcomes (BI and mRS scores) in patients with acute ischemic stroke. Further confirmation is required, but these results suggest that the intensity and severity of neurological deficits could be a good prediction of and explanation for the patient's ability to perform basic activities independently. Thus, physicians and therapists should consider neurological, motor and cognitive factors to improve patients' functional performance in stroke rehabilitation programs. Moreover, ADL tests could be used as valid tests for estimating the neurological impairment extent and recovery prediction in stroke victims.

## Future perspective

This study's findings provide the landmark for better exploration of the stroke risk factors, clinical characteristics and stroke scales of patients with acute ischemic stroke in a developing Mediterranean country with limited health resources. The study findings would be essential in the multidisciplinary health approach to update our knowledge, increase awareness, determine the high-risk subgroups and adequately plan health programs. Also, this study emphasizes the cornerstone roles of lifestyle modifications, medication compliance and physical exercise in strict controlling of stroke risk factors. The study recommendations would help achieve the desired health quality and rehabilitation plans and, thus, reduce ischemic stroke incidence and complications. Also, by this study, the authors tried to fill the literature gap regarding such issues outside Western countries, which would contribute to constructing global health guidelines. Future, more extensive, prospective, well-designed studies with longer follow-up are recommended to provide more data on the predictors of mortality and functional disability, and the determinative properties of stroke deficits for final functional outcomes in this population. Future clinical trials are needed to evaluate the potential appropriate interventions to improve the outcomes and reduce the morbidity and mortality rates in patients with ischemic stroke.

**Summary points**

- The prevalence estimates of stroke in low- and middle-income countries have doubled over the last 40 years, resulting in considerable disability and mortality rates, with an expected rise in the global burden of stroke.
- This study explored the demographics, clinical characteristics and stroke risk factors among the survivors of acute ischemic stroke at a tertiary university hospital in the north of Jordan. Also, it investigated the National Institutes of Health Stroke Scale (NIHSS), modified Rankin Scale (mRS) and Barthel Index (BI) scale scores at different times, their differences by the patients' characteristics and the correlations among these scales.
- Out of 376 screened patients with stroke, 359 survivors were included in the final analysis, with a mean (standard deviation) age of 67.8 (12.2) years.
- In this cohort, the men were at higher risk for acute ischemic stroke occurrence than the women (56.2% vs 43.8%, respectively).
- Compared with the male participants, the female participants scored significantly worse on the NIHSS at admission (7.61 [5.51] vs 9.47 [6.64]; p = 0.048, respectively), on the NIHSS at discharge (5.57 [4.72] vs 7.40 [5.88]; p = 0.028, respectively) and on the BI scale 1 month post-stroke event (78.68 [28.33] vs 66.03 [35.86]; p = 0.011, respectively). The mean (standard deviation) mRS score 1 month post-event was lower in men (2.4 [1.7]) than in women (2.9 [1.9]), but this difference was not statistically significant (p = 0.097).
- Thus, despite the male predominance in this cohort, the women tended to have a more severe stroke, worse neurological deficits, poorer functional outcomes and more significant disabilities than the men.
- Dysarthria was the most common speech abnormality (75.8%).
- Hyperlipidemia and hypertension (HNT) were the most prevalent stroke risk factors in the cohort, with prevalence estimates of 91.1% and 80.5%, respectively, higher than reported rates in the literature review.
- Hyperlipidemia had the highest sensitivity, PPV and NPV values among stroke risk factors.
- There were no statistically significant differences in the NIHSS, BI and mRS scores by comorbidities, including HTN, diabetes mellitus, HTN + diabetes mellitus, hyperlipidemia and atrial fibrillation (p > 0.05).
- Significant and strong correlations were observed between the NIHSS scores at admission and discharge, and BI and mRS scores 1 month after the stroke event. Thus, the authors concluded that neurological deficit severity has a potential role in predicting functioning and independence in performing ADLs outcomes and *vice versa*.
- A multicenter, prospective study on a large sample of patients with ischemic stroke investigating all potential stroke risk factors over a follow-up period of at least 12 months is suggested.

## References

[1] Warlow CP. Epidemiology of stroke. Lancet (London, UK) 352(Suppl. 3), Siii–S4 (1998). 10.1016/s0140-6736(98)90086-1 9803954

[2] Feigin VL, Nichols E, Alam T Global, regional, and national burden of neurological disorders, 1990–2016: a systematic analysis for the Global Burden of Disease Study 2016. Lancet Neurol. 18(5), 459–480 (2019). 30879893 10.1016/S1474-4422(18)30499-X PMC6459001

[3] Owolabi MO, Sarfo F, Akinyemi R Dominant modifiable risk factors for stroke in Ghana and Nigeria (SIREN): a case–control study. Lancet Glob. Health. 6(4), e436–e446 (2018). 29496511 10.1016/S2214-109X(18)30002-0 PMC5906101

[4] Cui Q, Naikoo NA. Modifiable and non-modifiable risk factors in ischemic stroke: a meta-analysis. Afr. Health Sci. 19(2), 2121–2129 (2019). 31656496 10.4314/ahs.v19i2.36 PMC6794552

[5] Gan Y, Wu J, Zhang S Prevalence and risk factors associated with stroke in middle-aged and older Chinese: a community-based cross-sectional study. Sci Rep. 7(1), 9501 (2017). 28842623 10.1038/s41598-017-09849-z PMC5572736

[6] Barker-Collo S, Bennett DA, Krishnamurthi RV Sex differences in stroke incidence, prevalence, mortality and disability-adjusted life years: results from the Global Burden of Disease Study 2013. Neuroepidemiology. 45(3), 203–214 (2015). 26505984 10.1159/000441103 PMC4632242

[7] Boehme AK, Esenwa C, Elkind MS. Stroke risk factors, genetics, and prevention. Circ. Res. 120(3), 472–495 (2017). 28154098 10.1161/CIRCRESAHA.116.308398 PMC5321635

[8] Sachdev PS, Lo JW, Crawford JD STROKOG (Stroke and Cognition Consortium): an international consortium to examine the epidemiology, diagnosis, and treatment of neurocognitive disorders in relation to cerebrovascular disease. Alzheimers Dement. (Amst). 7, 11–23 (2017). 28138511 10.1016/j.dadm.2016.10.006 PMC5257024

[9] Rawashdeh SI, Al-Mistarehi AH, Yassin A, Rabab'ah W, Skaff H, Ibdah R. A concurrent ischemic stroke, myocardial infarction, and aortic thrombi in a young patient with hyperhomocysteinemia: a case report. Int. Med. Case Rep. J. 13, 581–590 (2020). 33192104 10.2147/IMCRJ.S279603 PMC7653271

[10] Shah RS, Cole JW. Smoking and stroke: the more you smoke the more you stroke. Expert Rev. Cardiovasc. Ther. 8(7), 917–932 (2010). 20602553 10.1586/erc.10.56 PMC2928253

[11] Zhang FL, Guo ZN, Wu YH Prevalence of stroke and associated risk factors: a population based cross sectional study from northeast China. BMJ Open 7(9), e015758 (2017). 10.1136/bmjopen-2016-015758 PMC5589000 28871014

[12] Hakim AM. Depression, strokes and dementia: new biological insights into an unfortunate pathway. Cardiovasc. Psychiatry Neurol. 2011, 649629 (2011). 22216404 10.1155/2011/649629 PMC3246693

[13] Johnson W, Onuma O, Owolabi M, Sachdev S. Stroke: a global response is needed. Bull WHO 94(9), 634–634a (2016). 27708464 10.2471/BLT.16.181636 PMC5034645

[14] Benjamin EJ, Muntner P, Alonso A Heart disease and stroke statistics – 2019 update: a report from the American Heart Association. Circulation 139(10), e56–e528 (2019). 30700139 10.1161/CIR.0000000000000659

[15] Chauhan G, Debette S. Genetic risk factors for ischemic and hemorrhagic stroke. Curr. Cardiol. Rep. 18(12), 124 (2016). 27796860 10.1007/s11886-016-0804-z PMC5086478

[16] El-Hajj M, Salameh P, Rachidi S, Hosseini H. The epidemiology of stroke in the Middle East. Eur. Stroke J. 1(3), 180–198 (2016). 31008279 10.1177/2396987316654338 PMC6453228

[17] Tran J, Mirzaei M, Anderson L, Leeder SR. The epidemiology of stroke in the Middle East and North Africa. J. Neurol. Sci. 295(1-2), 38–40 (2010). 20541222 10.1016/j.jns.2010.05.016

[18] Robert AA, Zamzami MM. Stroke in Saudi Arabia: a review of the recent literature. Pan. Afr. Med. J. 17, 14 (2014). 10.11604/pamj.2014.17.14.3015 PMC4048673 24932325

[19] Alhazzani AA, Mahfouz AA, Abolyazid AY Study of stroke incidence in the Aseer region, southwestern Saudi Arabia. Int. J. Environ. Res. Pub. Health 15(2), 215 (2018). 10.3390/ijerph15020215 PMC5858284 29373563

[20] Fischer U, Arnold M, Nedeltchev K NIHSS score and arteriographic findings in acute ischemic stroke. Stroke 36(10), 2121–2125 (2005). 16151026 10.1161/01.STR.0000182099.04994.fc

[21] Ghandehari K. Challenging comparison of stroke scales. J. Res. Med. Sci. 18(10), 906–910 (2013). 24497865 PMC3897078

[22] Quinn TJ, Dawson J, Walters MR, Lees KR. Variability in modified Rankin scoring across a large cohort of international observers. Stroke 39(11), 2975–2979 (2008). 18688002 10.1161/STROKEAHA.108.515262

[23] Saver JL, Filip B, Hamilton S Improving the reliability of stroke disability grading in clinical trials and clinical practice: the Rankin Focused Assessment (RFA). Stroke 41(5), 992–995 (2010). 20360551 10.1161/STROKEAHA.109.571364 PMC2930146

[24] Lyden PD, Lu M, Levine SR, Brott TG, Broderick J. A modified National Institutes of Health Stroke Scale for use in stroke clinical trials: preliminary reliability and validity. Stroke 32(6), 1310–1317 (2001). 11387492 10.1161/01.str.32.6.1310

[25] Lyden P, Lu M, Jackson C Underlying structure of the National Institutes of Health Stroke Scale: results of a factor analysis. NINDS tPA Stroke Trial Investigators. Stroke 30(11), 2347–2354 (1999). 10548669 10.1161/01.str.30.11.2347

[26] Lyden P, Claesson L, Havstad S, Ashwood T, Lu M. Factor analysis of the National Institutes of Health Stroke Scale in patients with large strokes. Arch. Neurol. 61(11), 1677–1680 (2004). 15534178 10.1001/archneur.61.11.1677

[27] Adams HP Jr, Davis PH, Leira EC Baseline NIH Stroke Scale score strongly predicts outcome after stroke: a report of the Trial of Org 10172 in Acute Stroke Treatment (TOAST). Neurology 53(1), 126–131 (1999). 10408548 10.1212/wnl.53.1.126

[28] Demchuk AM, Tanne D, Hill MD Predictors of good outcome after intravenous tPA for acute ischemic stroke. Neurology 57(3), 474–480 (2001). 11502916 10.1212/wnl.57.3.474

[29] Mahoney FI, Barthel DW. Functional evaluation: the Barthel Index. Md. State Med. J. 14, 61–65 (1965). 14258950

[30] Collin C, Wade DT, Davies S, Horne V. The Barthel ADL Index: a reliability study. Int. Disabil. Stud. 10(2), 61–63 (1988). 3403500 10.3109/09638288809164103

[31] Granger CV, Hamilton BB, Gresham GE, Kramer AA. The stroke rehabilitation outcome study: Part II. Relative merits of the total Barthel Index score and a four-item subscore in predicting patient outcomes. Arch. Phys. Med. Rehabil. 70(2), 100–103 (1989). 2916925

[32] Broderick JP, Adeoye O, Elm J. Evolution of the modified Rankin Scale and its use in future stroke trials. Stroke 48(7), 2007–2012 (2017). 28626052 10.1161/STROKEAHA.117.017866 PMC5552200

[33] Quinn TJ, Dawson J, Walters MR, Lees KR. Reliability of the modified Rankin Scale: a systematic review. Stroke 40(10), 3393–3395 (2009). 19679846 10.1161/STROKEAHA.109.557256

[34] Von Elm E, Altman DG, Egger M, Pocock SJ, Gøtzsche PC, Vandenbroucke JP. The Strengthening the Reporting of Observational Studies in Epidemiology (STROBE) statement: guidelines for reporting observational studies. Ann. Intern. Med. 147(8), 573–577 (2007). 17938396 10.7326/0003-4819-147-8-200710160-00010

[35] Feigin VL, Forouzanfar MH, Krishnamurthi R Global and regional burden of stroke during 1990–2010: findings from the Global Burden of Disease Study 2010. Lancet (London, UK) 383(9913), 245–254 (2014). 10.1016/s0140-6736(13)61953-4 PMC4181600 24449944

[36] Soliman RH, Oraby MI, Fathy M, Essam AM. Risk factors of acute ischemic stroke in patients presented to Beni-Suef University Hospital: prevalence and relation to stroke severity at presentation. Egypt J. Neurol. Psychiatr. Neurosurg. 54(1), 8 (2018). 29780228 10.1186/s41983-018-0012-4 PMC5954772

[37] Grau AJ, Weimar C, Buggle F Risk factors, outcome, and treatment in subtypes of ischemic stroke: the German stroke data bank. Stroke 32(11), 2559–2566 (2001). 11692017 10.1161/hs1101.098524

[38] Altafi D, Khotbesara MH, Khotbesara MH, Bagheri A. A comparative study of NIHSS between ischemic stroke patients with and without risk factors. Tech. J. Eng. App. Sci. 3(17), 1954–1957 (2013).

[39] Deleu D, Inshasi J, Akhtar N Risk factors, management and outcome of subtypes of ischemic stroke: a stroke registry from the Arabian Gulf. J. Neurol. Sci. 300(1-2), 142–147 (2011). 20875650 10.1016/j.jns.2010.08.023

[40] El Tallawy HN, Farghaly WM, Badry R Epidemiology and clinical presentation of stroke in Upper Egypt (desert area). Neuropsychiatr. Dis. Treat. 11, 2177–2183 (2015). 26346729 10.2147/NDT.S87381 PMC4552260

[41] Yousufuddin M, Young N. Aging and ischemic stroke. Aging (Albany NY). 11(9), 2542–2544 (2019). 31043575 10.18632/aging.101931 PMC6535078

[42] Röther J, Alberts MJ, Touzé E Risk factor profile and management of cerebrovascular patients in the REACH Registry. Cerebrovasc. Dis. 25(4), 366–374 (2008). 18337635 10.1159/000120687

[43] Sacco RL, Diener HC, Yusuf S Aspirin and extended-release dipyridamole versus clopidogrel for recurrent stroke. New Engl. J. Med. 359(12), 1238–1251 (2008). 18753638 10.1056/NEJMoa0805002 PMC2714259

[44] ElAli A, Doeppner TR, Zechariah A, Hermann DM. Increased blood–brain barrier permeability and brain edema after focal cerebral ischemia induced by hyperlipidemia: role of lipid peroxidation and calpain-1/2, matrix metalloproteinase-2/9, and RhoA overactivation. Stroke 42(11), 3238–3244 (2011). 21836084 10.1161/STROKEAHA.111.615559

[45] Leppälä JM, Virtamo J, Fogelholm R, Albanes D, Heinonen OP. Different risk factors for different stroke subtypes: association of blood pressure, cholesterol, and antioxidants. Stroke 30(12), 2535–2540 (1999). 10582974 10.1161/01.str.30.12.2535

[46] Ayata C, Shin HK, Dileköz E Hyperlipidemia disrupts cerebrovascular reflexes and worsens ischemic perfusion defect. J. Cereb. Blood Flow Metab. 33(6), 954–962 (2013). 23486293 10.1038/jcbfm.2013.38 PMC3677117

[47] Carandang R, Seshadri S, Beiser A Trends in incidence, lifetime risk, severity, and 30-day mortality of stroke over the past 50 years. JAMA 296(24), 2939–2946 (2006). 17190894 10.1001/jama.296.24.2939

[48] Jimenez-Conde J, Biffi A, Rahman R Hyperlipidemia and reduced white matter hyperintensity volume in patients with ischemic stroke. Stroke 41(3), 437–442 (2010). 20133919 10.1161/STROKEAHA.109.563502 PMC3787512

[49] Shigematsu K, Watanabe Y, Nakano H. Influences of hyperlipidemia history on stroke outcome; a retrospective cohort study based on the Kyoto Stroke Registry. BMC Neurol. 15, 44 (2015). 25880411 10.1186/s12883-015-0297-1 PMC4376998

[50] Arsava EM, Rahman R, Rosand J Severity of leukoaraiosis correlates with clinical outcome after ischemic stroke. Neurology 72(16), 1403–1410 (2009). 19380699 10.1212/WNL.0b013e3181a18823 PMC2677507

[51] Cohen JI, Cazettes F, Convit A. Abnormal cholesterol is associated with prefrontal white matter abnormalities among obese adults: a diffusion tensor imaging study. Neuroradiol. J. 24(6), 854–861 (2011). 24059886 10.1177/197140091102400604

[52] Restrepo L, Bang OY, Ovbiagele B Impact of hyperlipidemia and statins on ischemic stroke outcomes after intra-arterial fibrinolysis and percutaneous mechanical embolectomy. Cerebrovasc. Dis. 28(4), 384–390 (2009). 19713698 10.1159/000235625 PMC2909704

[53] Xing Y, An Z, Yu N, Zhao W, Ning X, Wang J. Low density lipoprotein cholesterol and the outcome of acute ischemic stroke: results of a large hospital-based study. Eur. Neurol. 76(5-6), 195–201 (2016). 27705971 10.1159/000450604

[54] Amarenco P, Lavallée P, Touboul PJ. Stroke prevention, blood cholesterol, and statins. Lancet Neurol. 3(5), 271–278 (2004). 15099541 10.1016/S1474-4422(04)00734-3

[55] Bedi A, Flaker GC. How do HMG-CoA reductase inhibitors prevent stroke? Am. J. Cardiovasc. Drugs 2(1), 7–14 (2002). 14727994 10.2165/00129784-200202010-00002

[56] Endres M. Statins and stroke. J. Cereb. Blood Flow Metab. 25(9), 1093–1110 (2005). 15815580 10.1038/sj.jcbfm.9600116

[57] Amarenco P, Bogousslavsky J, Callahan A 3rd High-dose atorvastatin after stroke or transient ischemic attack. New Engl. J. Med. 355(6), 549–559 (2006). 16899775 10.1056/NEJMoa061894

[58] Rodríguez-García JL, Botia E, de La Sierra A, Villanueva MA, González-Spínola J. Significance of elevated blood pressure and its management on the short-term outcome of patients with acute ischemic stroke. Am. J. Hypertens. 18(3), 379–384 (2005). 15797657 10.1016/j.amjhyper.2004.10.004

[59] Pathak A, Kumar P, Pandit AK Is prevalence of hypertension increasing in first-ever stroke patients? A hospital-based cross-sectional study. Ann Neurosci. 25(4), 219–222 (2018). 31000960 10.1159/000487066 PMC6470354

[60] Qureshi AI, Ezzeddine MA, Nasar A Prevalence of elevated blood pressure in 563,704 adult patients with stroke presenting to the ED in the United States. Am. J. Emerg. Med. 25(1), 32–38 (2007). 17157679 10.1016/j.ajem.2006.07.008 PMC2443694

[61] Jing L, Tian Y, Ren G Epidemiological features of hypertension among ischemic survivors in northeast China: insights from a population-based study, 2017–2019. BMC Pub. Health 21(1), 1648 (2021). 34503467 10.1186/s12889-021-11692-x PMC8427863

[62] Essa AYE, Helmy TA, Batch SSAE. Study of incidence, risk factors and outcome of acute cerebrovascular stroke patients admitted to Alexandria Main University Hospital. J. Am. Sci. 7(11), 316–329 (2011).

[63] Saposnik G, Gladstone D, Raptis R, Zhou L, Hart RG. Atrial fibrillation in ischemic stroke: predicting response to thrombolysis and clinical outcomes. Stroke 44(1), 99–104 (2013). 23168456 10.1161/STROKEAHA.112.676551

[64] Kim K. Relation of stroke risk factors to severity and disability after ischemic stroke. Korean J. Stroke 14(3), 136–141 (2012).

[65] Caso V, Paciaroni M, Agnelli G Gender differences in patients with acute ischemic stroke. Women's Health 6(1), 51–57 (2010). 10.2217/whe.09.82 20088729

[66] Eriksson M, Glader EL, Norrving B, Terént A, Stegmayr B. Sex differences in stroke care and outcome in the Swedish national quality register for stroke care. Stroke 40(3), 909–914 (2009). 19118246 10.1161/STROKEAHA.108.517581

[67] Appelros P, Stegmayr B, Terént A. Sex differences in stroke epidemiology: a systematic review. Stroke 40(4), 1082–1090 (2009). 19211488 10.1161/STROKEAHA.108.540781

[68] Di Carlo A, Lamassa M, Baldereschi M Sex differences in the clinical presentation, resource use, and 3-month outcome of acute stroke in Europe: data from a multicenter multinational hospital-based registry. Stroke 34(5), 1114–1119 (2003). 12690218 10.1161/01.STR.0000068410.07397.D7

[69] Elkind MSV, Prabhakaran S, Pittman J, Koroshetz W, Jacoby M, Johnston KC. Sex as a predictor of outcomes in patients treated with thrombolysis for acute stroke. Neurology 68(11), 842–848 (2007). 17353472 10.1212/01.wnl.0000256748.28281.ad

[70] Smith MA, Lisabeth LD, Brown DL, Morgenstern LB. Gender comparisons of diagnostic evaluation for ischemic stroke patients. Neurology 65(6), 855–858 (2005). 16186523 10.1212/01.wnl.0000176054.72325.0f

[71] Foerch C, Misselwitz B, Humpich M, Steinmetz H, Neumann-Haefelin T, Sitzer M. Sex disparity in the access of elderly patients to acute stroke care. Stroke 38(7), 2123–2126 (2007). 17525398 10.1161/STROKEAHA.106.478495

[72] Musa KI, Keegan TJ. The change of Barthel Index scores from the time of discharge until 3-month post-discharge among acute stroke patients in Malaysia: a random intercept model. PLOS ONE 13(12), e0208594 (2018). 30571691 10.1371/journal.pone.0208594 PMC6301695

[73] Phan TG, Clissold BB, Ma H, Ly JV, Srikanth V. Predicting disability after ischemic stroke based on comorbidity index and stroke severity – From the Virtual International Stroke Trials Archive – acute collaboration. Front. Neurol. 8, 192 (2017). 28579970 10.3389/fneur.2017.00192 PMC5437107

[74] Fischer U, Arnold M, Nedeltchev K Impact of comorbidity on ischemic stroke outcome. Acta Neurol. Scand. 113(2), 108–113 (2006). 16411971 10.1111/j.1600-0404.2005.00551.x

[75] Khassawneh B, Ibnian A, Yassin A The outcome of patients with acute stroke requiring intensive care unit admission. Eur. Respir. J. 54(Suppl. 63), PA2283 (2019).

[76] Wouters A, Nysten C, Thijs V, Lemmens R. Prediction of outcome in patients with acute ischemic stroke based on initial severity and improvement in the first 24 h. Front. Neurol. 9, 308 (2018). 29867722 10.3389/fneur.2018.00308 PMC5950843

[77] Sablot D, Belahsen F, Vuillier F Predicting acute ischaemic stroke outcome using clinical and temporal thresholds. ISRN Neurol. 2011, 354642 (2011). 22462018 10.5402/2011/354642 PMC3302020

[78] Goldstein LB, Samsa GP. Reliability of the National Institutes of Health Stroke Scale. Extension to non-neurologists in the context of a clinical trial. Stroke 28(2), 307–310 (1997). 9040680 10.1161/01.str.28.2.307

[79] Akbari S, Lyden PD, Kamali M, Fahimi MA. Correlations among impairment, daily activities and thinking operations after stroke. NeuroRehabilitation 33(1), 153–160 (2013). 23949044 10.3233/NRE-130940

[80] Akbari S, Ashayeri H, Fahimi MA, Kamali M, Lyden PD. The correlation of independency in activities of daily living performance with cognitive status and the intensity of neurological impairment in right-handed stroke patients. NeuroRehabilitation 29(3), 311–316 (2011). 22142765 10.3233/NRE-2011-0707

[81] Kwakkel G, Veerbeek JM, van Wegen EE, Nijland R, Harmeling-van der Wel BC, Dippel DW. Predictive value of the NIHSS for ADL outcome after ischemic hemispheric stroke: does timing of early assessment matter? J. Neurol. Sci. 294(1-2), 57–61 (2010). 20439108 10.1016/j.jns.2010.04.004

[82] Saver JL, Altman H. Relationship between neurologic deficit severity and final functional outcome shifts and strengthens during first hours after onset. Stroke 43(6), 1537–1541 (2012). 22492517 10.1161/STROKEAHA.111.636928 PMC3509751

[83] Kong KH, Lee J. Temporal recovery of activities of daily living in the first year after ischemic stroke: a prospective study of patients admitted to a rehabilitation unit. NeuroRehabilitation 35(2), 221–226 (2014). 24990018 10.3233/NRE-141110

[84] Al-Mistarehi A-H, Elsayed MA, Ibrahim RM Clinical outcomes of primary subarachnoid hemorrhage: an exploratory cohort study from Sudan. Neurohospitalist 12(2), 249–263 (2022). 35419154 10.1177/19418744211068289 PMC8995598

[85] Naghavi FS, Koffman EE, Lin B, Du J. Post-stroke neuronal circuits and mental illnesses. Int. J. Physiol. Pathophysiol. Pharmacol. 11(1), 1–11 (2019). 30911356 PMC6420715

[86] Rawashdeh SI, Ibdah R, Kheirallah KA Prevalence estimates, severity, and risk factors of depressive symptoms among coronary artery disease patients after ten days of percutaneous coronary intervention. Clin. Pract. Epidemiol. Ment. Health 17, 103–113 (2021). 34733349 10.2174/1745017902117010103 PMC8493832

[87] Khassawneh AH, Alzoubi A, Khasawneh AG The relationship between depression and metabolic control parameters in Type 2 diabetic patients: a cross-sectional and feasibility interventional study. Int. J. Clin. Pract. 75(4), e13777 (2021). 33098211 10.1111/ijcp.13777

[88] Beloosesky Y, Streifler JY, Burstin A, Grinblat J. The importance of brain infarct size and location in predicting outcome after stroke. Age Ageing 24(6), 515–518 (1995). 8588543 10.1093/ageing/24.6.515

[89] Yassin A, Ghzawi A, Al-Mistarehi AH Mortality rate and biomarker expression within COVID-19 patients who develop acute ischemic stroke: a systematic review and meta-analysis. Future Sci OA. 7(7), Fso713 (2021). 34254031 10.2144/fsoa-2021-0036 PMC8114837

[90] El-Salem K, Al-Mistarehi AH, Khalil H, Al-Sharman A, Yassin A. Serum tumor necrosis factor-alpha levels correlate with cognitive function scales scores in multiple sclerosis patients. Mult. Scler. Relat. Disord. 47, 102621 (2021). 33197871 10.1016/j.msard.2020.102621

[91] El-Salem K, Khalil H, Al-Sharman A Serum vitamin d inversely correlates with depression scores in people with multiple sclerosis. Mult. Scler. Relat. Disord. 48, 102732 (2021). 33422916 10.1016/j.msard.2020.102732

[92] Wallace M, Shelkey M. Katz Index of Independence in Activities of Daily Living(ADL). Urologic Nursing 27(1), 93–94 (2007). 17390935

[93] Sarker SJ, Rudd AG, Douiri A, Wolfe CD. Comparison of 2 extended activities of daily living scales with the Barthel Index and predictors of their outcomes: cohort study within the South London Stroke Register (SLSR). Stroke 43(5), 1362–1369 (2012). 22461336 10.1161/STROKEAHA.111.645234

[94] Tsang RC, Chau RM, Cheuk TH The measurement properties of modified Rivermead mobility index and modified functional ambulation classification as outcome measures for Chinese stroke patients. Physiother. Theory Pract. 30(5), 353–359 (2014). 24400683 10.3109/09593985.2013.876563

[95] Lindmark B. Evaluation of functional capacity after stroke with special emphasis on motor function and activities of daily living. Scand. J. Rehabil. Med. Suppl. 21, 1–40 (1988). 3071845

